# Spatial pattern of the population casualty rate caused by super typhoon Lekima and quantification of the interactive effects of potential impact factors

**DOI:** 10.1186/s12889-021-11281-y

**Published:** 2021-06-29

**Authors:** Xiangxue Zhang, Juan Nie, Changxiu Cheng, Chengdong Xu, Xiaojun Xu, Bin Yan

**Affiliations:** 1 Key Laboratory of Environmental Change and Natural Disaster, Ministry of Education, Beijing Normal University, Beijing, 100875 China; 2 State Key Laboratory of Resources and Environmental Information System, Institute of Geographic Sciences and Natural Resources Research, Chinese Academy of Sciences, Beijing, 100101 China; 3 National Disaster Reduction Center of China, Ministry of Emergency Management, Beijing, 100124 China; 4 State Key Laboratory of Earth Surface Processes and Resource Ecology, Beijing Normal University, Beijing, 100875 China; 5 National Tibetan Plateau Data Center, Beijing, 100101 China; 6 College of Forestry and Landscape Architecture, South China Agricultural University, Guangzhou, 510642 China

**Keywords:** GeoDetector, Lekima, Spatial pattern, Population casualty, Interactive effects

## Abstract

**Background:**

Typhoons greatly threaten human life and property, especially in China. Therefore, it is important to make effective policy decisions to minimize losses associated with typhoons.

**Methods:**

In this study, the GeoDetector method was used to quantify the determinant powers of natural and socioeconomic factors, and their interactions, on the population casualty rate of super typhoon Lekima. The local indicator of spatial association (LISA) method was followed to explore the spatial pattern of the population casualty rate under the influence of the identified dominant factors.

**Results:**

Both natural and socioeconomic factors were found to have significantly impacted the population casualty rate due to super typhoon Lekima. Among the selected factors, maximum precipitation was dominant factor (*q* = 0.56), followed by maximum wind speed (*q* = 0.45). In addition, number of health technicians (*q* = 0.35) and number of health beds (*q* = 0.27) have a strong influence on the population casualty rate. Among the interactive effects of 12 influencing factors, the combined effects of maximum precipitation and ratio of brick-wood houses, the maximum precipitation and ratio of steel-concrete houses, maximum precipitation and number of health technicians were highest (*q* = 0.72). Furthermore, high-risk areas with very high casualty rates were concentrated in the southeastern part of Zhejiang and northern Shandong Provinces, while lower-risk areas were mainly distributed in northern Liaoning and eastern Jiangsu provinces.

**Conclusions:**

These results contribute to the development of more specific policies aimed at safety and successful property protection according to the regional differences during typhoons.

## Background

Typhoons are among the most frequent and disastrous natural hazards in the world, inflicting great losses on human life and property, have affected the lives of more than 250 million people worldwide [1–4]. Typhoons are often accompanied by extreme weather, such as coastal erosion, storm surge and heavy precipitation that can lead to floods and landslides [3, 5]. These hazards should not be underestimated as they can result in significant loss of housing and infrastructure as well as posing a considerable threat to human life, especially in coastal areas [6–8]. China is currently among those countries most vulnerable to typhoons [9], particularly coastal areas of that.

In the previous studies, typhoon disaster risk commonly refers to the probability that a typhoon will cause harmful consequences or expected losses to elements at risk within a certain period of time, and is related to hazards, vulnerability, exposure, and disaster prevention and mitigation capabilities [10]. Among them, hazards represent a potentially destructive physical event, phenomenon, or human activity that may cause loss of life or injury, property loss, socioeconomic chaos, and environmental degradation, such as the hazards of typhoon disasters include storm surge, heavy rainfall, landslides (debris flow), coastal erosion, and so on [3, 5]. Vulnerability represents how exposed entities are affected by typhoon disasters, in which personnel vulnerability refers to the characteristics that cause individuals or groups to be injured and died by typhoon disasters [11]. Exposure refers to the number of people or buildings exposed to and adversely affected by typhoon disasters [12]. The ability of disaster prevention and mitigation represents how an area can effectively recover from short-term and long-term impacts caused by the typhoon disaster. It is the ability of the area to prevent and respond to typhoon disasters and recover from disasters. Therefore, the study of typhoon disaster risk must comprehensively consider the hazard, vulnerability, exposure and disaster prevention and mitigation capabilities, so as to provide reference and basis for the decision-making of resource allocation and disaster prevention and mitigation planning in a certain area.

That is, in a region, the magnitude of the risks with associated typhoons is not only dependent on their intensity, but also on the level of economic development, population density, the level of medical facilities, and human activities in the affected areas [13, 14]. With socioeconomic development, increasing numbers of people and property are becoming exposed to typhoons [14, 15]. Furthermore, with global warming, typhoon-induced losses in China may be higher in the future, particularly in southeastern coastal areas that most frequently experience typhoons, such as Zhejiang and Guangdong Provinces [4]. Therefore, the dominant factors resulting in risks during typhoons should be determined and recorded.

In recent years, previous research has demonstrated that the risk of typhoons is not only related to climate change parameters, such as wind speed, ocean temperature changes, the El Niño-Southern Oscillation, and sea-level rise [14, 16, 17], but also the vulnerability of hazard-affected bodies and capacity of disaster prevention and reduction. Notably, with urbanization, human socioeconomic activities will aggravate the impacts of natural disasters. Since the 1980s, research on typhoon disasters has increasingly focused on the safe construction of the economy and society, and has highlighted the importance of vulnerability, exposure, sensitivity, and adaptive capacity in economic, social, and cultural systems [18, 19]. This has become a multi-scale, comprehensive concept influenced by nature, society, economy, and the environment [11]. Therefore, the links between socioeconomic activities and disaster losses caused by typhoons have attracted widespread attention in a range of fields, including economics, urban studies, architecture, and the social sciences [18, 19].

Most previous research has focused on analyzing the impacts of single natural or socioeconomic factors on disaster risks [20, 21], while interactive effects are more rarely considered. Additionally, traditional statistical methods cannot detect interactive effects between those factors affecting disaster losses, as traditional regression methods typically consider the products of two individual factors [22]. Additionally, statistical models of interactive effects are usually created for local regions, and their ability to reflect broader spatial variability is limited. Coefficients with spatial differences can be derived using Geographical Information Systems (GIS)-based regression methods and machine learning algorithms, but these have poor large-scale explanatory capability due to the existence of spatially stratified heterogeneity [23, 24].

Regional and global typhoon risk management highlights the importance and necessity of studying the factors influencing typhoon disaster risks. The eastern coastal region of China is undergoing rapid urbanization, becoming an increasingly important metropolitan group. However, this region is also one of the main areas facing severe typhoon risks. Therefore, quantifying the determinant powers of impact factors and their interactive effects is important for successfully formulating policies to control and reduce casualties and property losses. In this study, focusing on the 2019 super typhoon Lekima, hazard factors, the sensitivity of the disaster environment, the vulnerability of hazard-affected bodies, and disaster prevention and mitigation are considered simultaneously. Specifically, this study aimed to (1) quantify the determinant powers and interactive effects of natural and socioeconomic factors on the population casualty rate resulting from super typhoon Lekima using the GeoDetector method; (2) identify the dominant factors affecting the population casualty rate; and (3) examine the spatial patterns of the population casualty rate under the influence of the dominant factor using the LISA method.

## Materials and methods

### Typhoon data

On August 10, 2019, super typhoon Lekima made landfall on Wenling City, Zhejiang Province, followed by Qingdao City, Shandong Province, data regarding the development and characteristics of the typhoon were obtained from the China Meteorological Administration (http://2011.cma.gov.cn), and data on total population of a county was obtained from Liaoning, Shandong, Jiangsu, and Zhejiang province governmental statistical yearbooks (https://data.cnki.net/Yearbook/Navi? type = type&code = A).

**Fig. 1:**
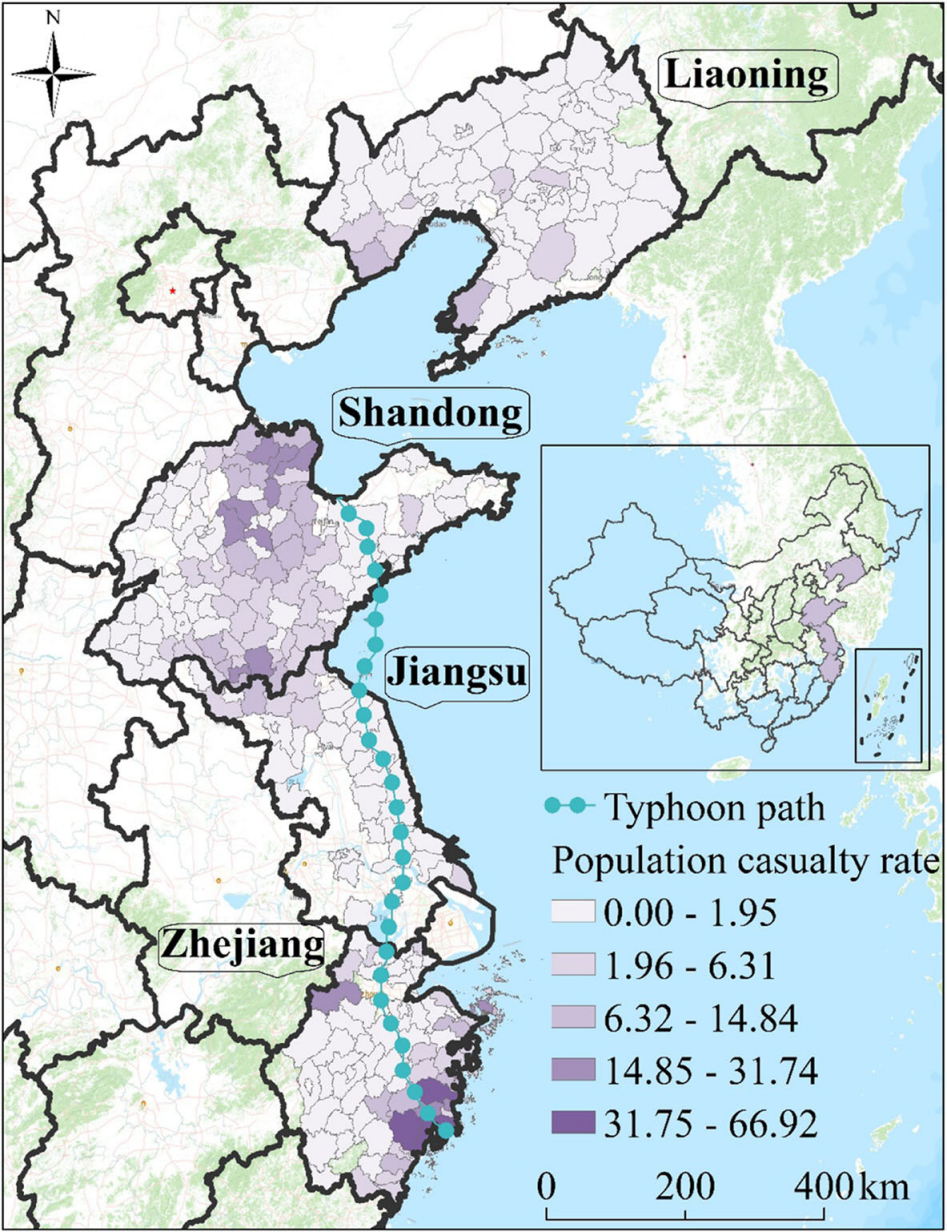
Geographic location of the study area and spatial distribution of population casualty rate attributable to super typhoon Lekima (The administrative map in the figure was obtained from the Resource and Environment Data Cloud Platform (http://www.resdc.cn))

Notably, according to records, super typhoon Lekima was the fifth strongest typhoon to hit mainland China since 1949, resulted in a total of 14.02 million population casualties, the collapse of 15,000 houses and the loss of 11.37 thousand hectares of agricultural land equating to direct economic losses of 51.53 billion CNY, in which the population casualty in this study represents the total number of people died and injured caused by super typhoon Lekima; the population casualty rate represents the ratio of total number of people died and injured caused by super typhoon Lekima divided by the total population in a county. Therefore, the spatial characteristics of the disaster for the four provinces most affected by Super Typhoon Lekima, namely, Zhejiang, Jiangsu, Shandong, and Liaoning are analyzed here, which are located in the eastern coastal region of China, and densely populated and economically well developed (Fig. 1). Moreover, this region spans a length of more than 18,000 km along the coastline and often suffer from extreme weather events, such as strong winds and heavy precipitation caused by typhoons, with such events typically resulting in significant numbers of casualties and property losses. Therefore, it is essential to deploy rapid emergency measures against typhoons in this eastern coastal area of China.

### Impact factors

**Fig. 2:**
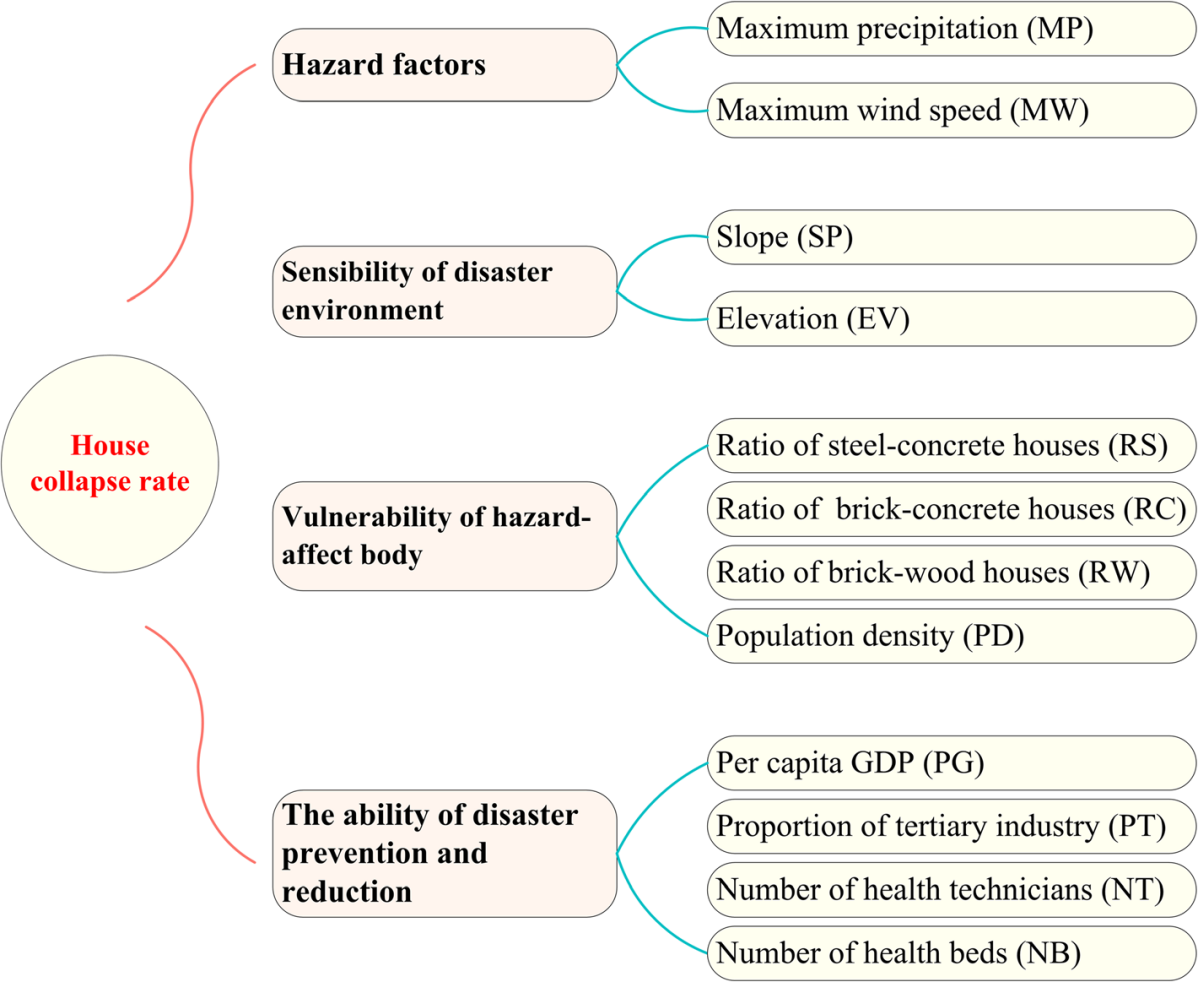
Proxy and potential impact factors

Considering hazard factors, sensibility of disaster environment, vulnerability of hazard-affected bodies, and ability of disaster prevention and reduction, results from previous studies [13, 18, 25, 26] and data availability, 12 natural and socioeconomic variables were selected in this study. Data collected during the same period from governmental statistic yearbooks of Zhejiang, Jiangsu, Shandong, and Liaoning Provinces (Fig. 2), including house structures, such as the ratio of steel-concrete houses (RS), ratio of brick-concrete houses (RC), and ratio of brick-wood houses (RW), were also considered. In addition, population density (PD), per capita gross domestic product (GDP) (PG), proportion of tertiary industry (PT), number of health beds (NB), number of health technicians (NT), daily maximum precipitation (MP), and maximum wind speed (MW) associated with Super Typhoon Lekima were also included. Data on the slope (SP) and elevation (EV) (resolution of 1 km × 1 km) were downloaded from the resource and environmental data cloud platform (http://www.resdc.cn). Then, the mean values of SP and EV were calculated using zonal statistics in ArcGIS 10.3 software for each county.

### Statistical analysis

In this study, the GeoDetector was used to quantify the determinant powers of single natural and socioeconomic factors and their interactive effects on the population casualty rate attributable to super typhoon Lekima, allowing the dominant factors to be determined. Then the local indicator of spatial association (LISA) was used to identify the spatial pattern of population casualty rate under the influence of the dominant factor, and further classify the study area into hot spots (high-risk areas) and cold spots (low-risk areas).

### GeoDetector

GeoDetector (www.geodetector.cn) is a suitable tool for handling the phenomenon with spatially stratified heterogeneity. The basic idea of GeoDetector is that if a factor X affects a dependent variable Y to a certain extent, the dependent variable Y will exhibit a spatial distribution similar to that of factor X [24, 27, 28]. This idea is more comprehensive than traditional methods and truly reflects geographical phenomena, has been widely used in disaster and health fields [29–31]. In this study, GeoDetector was introduced to quantify the relationships between the dependent variable (population casualty rate) and the natural and socioeconomic factors, here we classified the values of each impact variable into 6 levels, and the definition of *q* as follows:

1 $$ q=1-\frac{1}{N{\sigma}^2}{\sum}_{\mathrm{h}=1}^L{N}_h{\sigma}_h^2 $$

where *q* represents the degree to which the influence factor X explains the spatial heterogeneity of the dependent variable Y (population casualty rate), and its value ranges from 0 to 1. The larger the value of *q*, the stronger the effect of the independent variable X on the dependent variable Y. The study area was divided into *L* layers, which are denoted by *h* = 1, 2, ..., *L*. *N* and *N*_*h*_ are the number of counties in the entire study area and strata *h*, respectively. *σ*^*2*^ and *σ*^*2*^_*h*_ are the variances of the entire area and strata *h*, respectively.

**Fig. 3:**
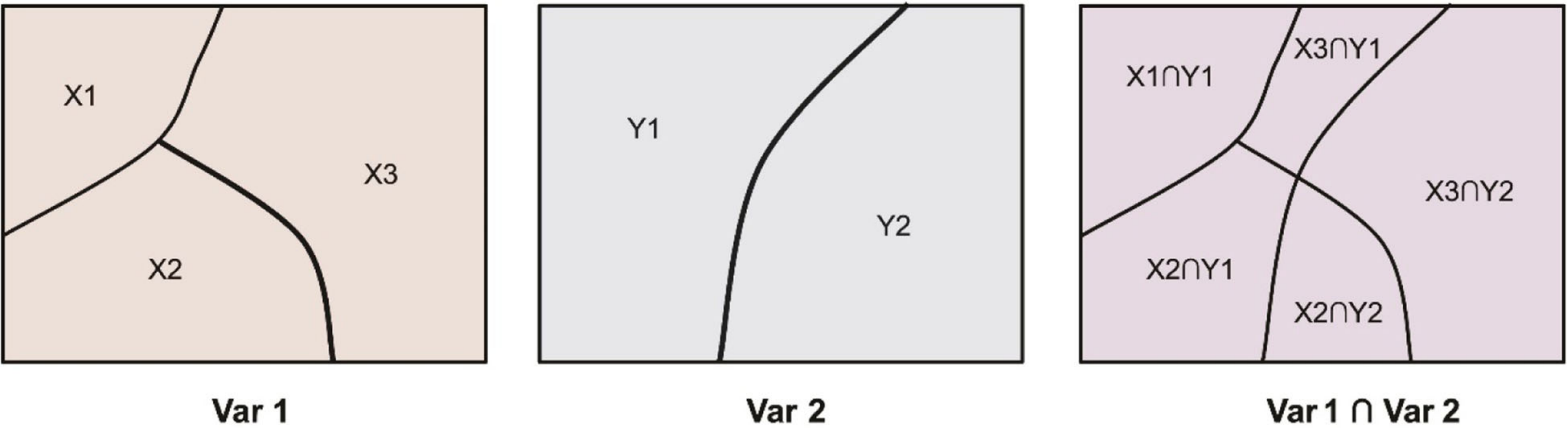
Principle of interaction detector

GeoDetector can also be used to identify interactive effects between two random impact factors to evaluate whether factors X1 and X2 work together to increase or decrease the determinant power of the single factor on the dependent variable Y, or whether these factors affecting Y are independent of each other. GeoDetector first calculate the *q* value of the two factors X1, X2, namely, *q* (X1), *q* (X2) and their interactive effects *q* (X1∩X2) (the new layer formed by the tangency of the two layers of the overlapping variable X1 and the X2 polygon distribution, Fig. 3), and then compare *q* (X1), *q* (X2) and *q* (X1∩X2).

It is worth mentioning that one of the advantages of GeoDetector is that the variables have no linear assumption, which means that the multicollinearity of the input factors can be ignored. Therefore, adding new factors or excluding existing factors will not affect the results of the other factors.

### Identification of spatial pattern

Bivariate spatial correlation analysis is an extension of spatial correlation analysis and can be used to examine the spatial correlation of bivariate observations [32]. Accordingly, the local Moran’s *I* value was used to identify the hot and cold spots for the population casualty rate and determine the class of spatial correlation between the two studied subjects, as follows:

2 $$ {I}_{kl}^i={Z}_k^i{\sum}_{j=l}^n{W}_{ij}{Z}_l^i $$

3 $$ {Z}_k^i=\frac{x_k^i-\overline{x_k}}{\sigma_k},{Z}_l^i=\frac{x_l^i-{\overline{x}}_l}{\sigma_l} $$

where *W*_*ij*_ is the spatial weight matrix, *x*_*k*_^*i*^ is the observation *k* at location *i*, *x*_*l*_^*j*^ is the observation *l* at location *j*, ${\overline{x}}_k$ and ${\overline{x}}_l$ are the mean values of *x*_*k*_ and *x*_*l*_, respectively, and *σ*_*k*_ and *σ*_*l*_ are the variances of *x*_*k*_ and *x*_*l*_, respectively.

*Z*_*k*_^*i*^ and the corresponding spatial lag *WZ*_*l*_^*i*^ at location *i* are presented on the vertical and horizontal axes of Moran’s *I* scatter plot, respectively [33]. The spatial correlation is then divided into four quadrants by the two-coordinate axis. The first and third quadrants indicate that the two variables in these spatial units have a positive spatial correlation or spatial clustering (High-High and Low-Low), in which spatial clustering is the value of two variables with significant positive spatial correlation in some spatial units. The second and fourth quadrants indicate that the bivariate variables in these spatial units have a negative spatial correlation or spatial outliers (High-Low and Low-High), in which spatial outliers are bivariate values that have significant negative spatial correlation in some spatial units [32]. All LISA calculations were performed in GeoDa.

## Results

### Detection of dominant factors

In this study, GeoDetector was used to quantify the determinant powers of the selected 12 natural and socioeconomic factors on the population casualty rate attributable to super typhoon Lekima, the importance of each factor sorted in descending order according to the *q* values of the impact factors (Table 1), and the dominant factors of spatial heterogeneity were further captured. The results showed that, among the selected variables, maximum precipitation had the strongest effect on population casualty rate, with a *q* value of 0.56, followed by maximum wind speed, with a *q* value of 0.45. This shows that heavy precipitation and strong wind induced by super typhoon Lekima had the strongest influences on human life.

**Table 1:** The *q* and *p* values of each influence factor

Influence factors	*q*	*p*
Maximum precipitation (mm)	0.56	0.00
Maximum wind speed (m/s)	0.45	0.00
Number of health technicians (per 10^3^)	0.35	0.00
Number of health beds (per 10^3^)	0.27	0.00
Per capita gross domestic product (GDP) (10^4^CNY)	0.16	0.01
Population density (10^4^person/km^2^)	0.14	0.03
Ratio of steel-concrete houses (100%)	0.14	0.00
Ratio of brick-wood houses (100%)	0.12	0.00
Ratio of brick-concrete houses (100%)	0.11	0.00
Slope (degrees)	0.08	0.04
Proportion of the tertiary industry (100%)	0.07	0.01
Elevation (m)	0.06	0.05

The socioeconomic factors, number of health technicians, number of health beds, per capita GDP and population density, also exhibited a significant correlation with population casualty rate. The *q* values of number of health technicians, number of health beds, per capita GDP and population density were 0.35, 0.27, 0.16 and 0.14, respectively, which indicates that the effects of economic level, medical level, and human activity are non-negligible in determining the population casualty rate caused by the super typhoon Lekima (Table 1).

The results also showed that house structures significantly impacted the population casualty rate during the arrival of the typhoon. For example, the *q* values of ratio of steel-concrete houses, ratio of brick-wood houses, and ratio of brick-concrete houses were 0.14, 0.12, and 0.11, respectively, showing that the difference in the house structures is also non-negligible for the population casualty rate attributable to the super typhoon Lekima (Table 1).

Slope and elevation also significantly impacted the population casualty rate, with *q* values of 0.08 and 0.06, respectively. This shows that natural environmental situations also impact on the population casualty when there hit by the typhoon (Table 1).

Proportion of tertiary industry also has a certain determinant power on the population casualty rate, with a *q* value of 0.07, which also shows that the economic level also has a non-negligible impact on the population casualty rate (Table 1).

### Interactive effects among the impact factors

**Fig. 4:**
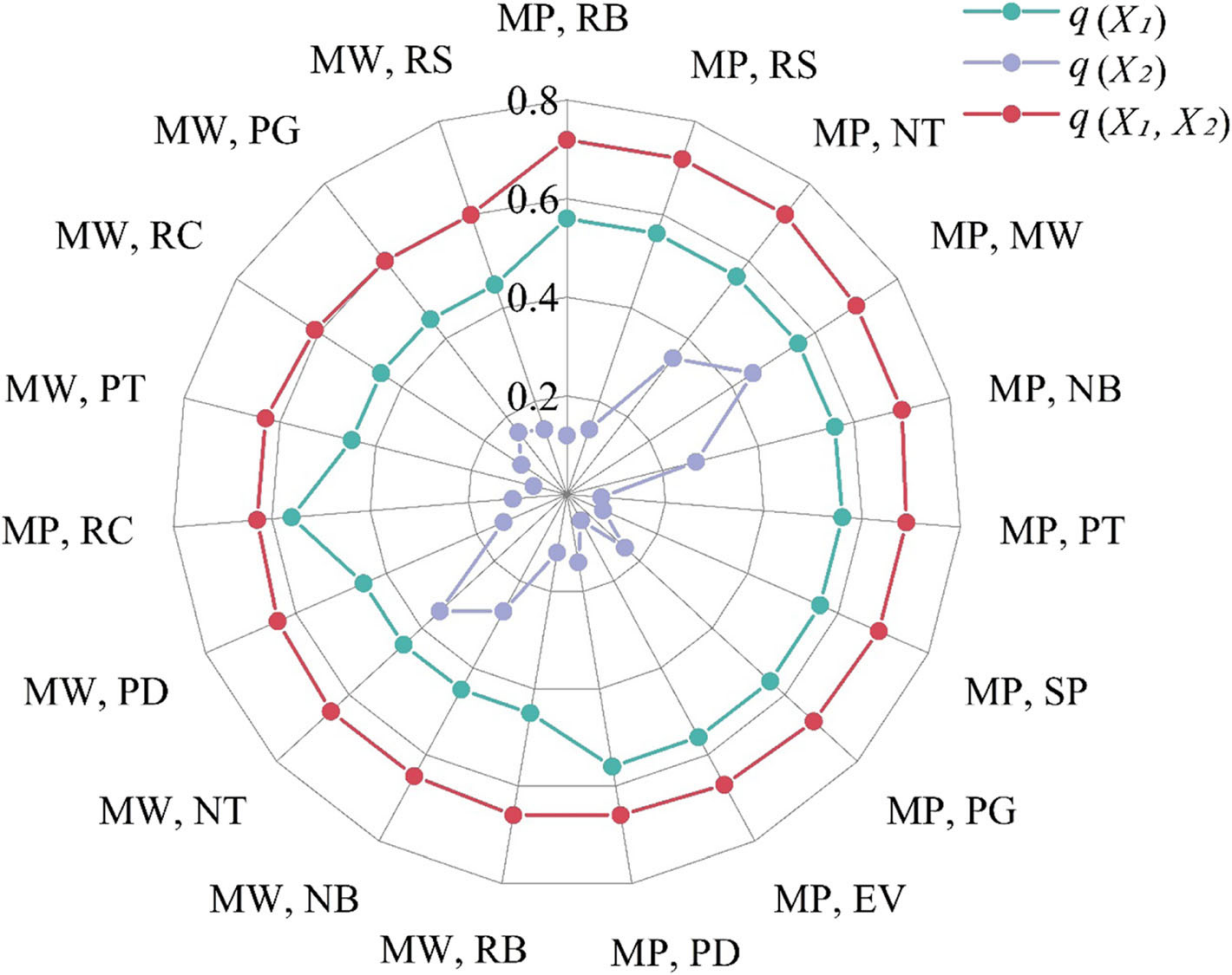
Interactive effects between natural and socioeconomic factors. Note: *X*_*1*_ represents the first factor, *X*_*2*_ denotes the second factor, and *X*_*1*_, *X*_*2*_ represents the interactive effect of the two random factors, in which has showed the *q* value of interactive effect greater than 0.60

A total of 66 pairs of interactive effects among the 12 factors were calculated using GeoDetector. Considering the results of the interactive effects among selected factors (Fig. 4), the interactive effects of each pair of factors were found to be significantly larger than the *q* value of the two factors individually. This indicates that the population casualty rate is not only affected by individual factors, but also by interactive effects between two random factors, with greater impact. According to the results of interactive effects of two random factors, the values of *q* (maximum precipitation ∩ ratio of brick-wood houses), *q* (maximum precipitation ∩ ratio of steel-concrete houses), and *q* (maximum precipitation ∩ number of health technicians) were the highest (*q* = 0.72), followed by *q* (maximum precipitation ∩ maximum wind speed) and *q* (maximum precipitation ∩ number of health beds) with *q* values both of 0.70. These results indicate that the interactive effects between maximum precipitation and house structures, such as houses of brick-wood and steel-concrete structures, is the strongest, and that the interactive effects between natural and socioeconomic factors are far stronger than the effects of individual natural or socioeconomic factors.

### Spatial pattern

**Fig. 5:**
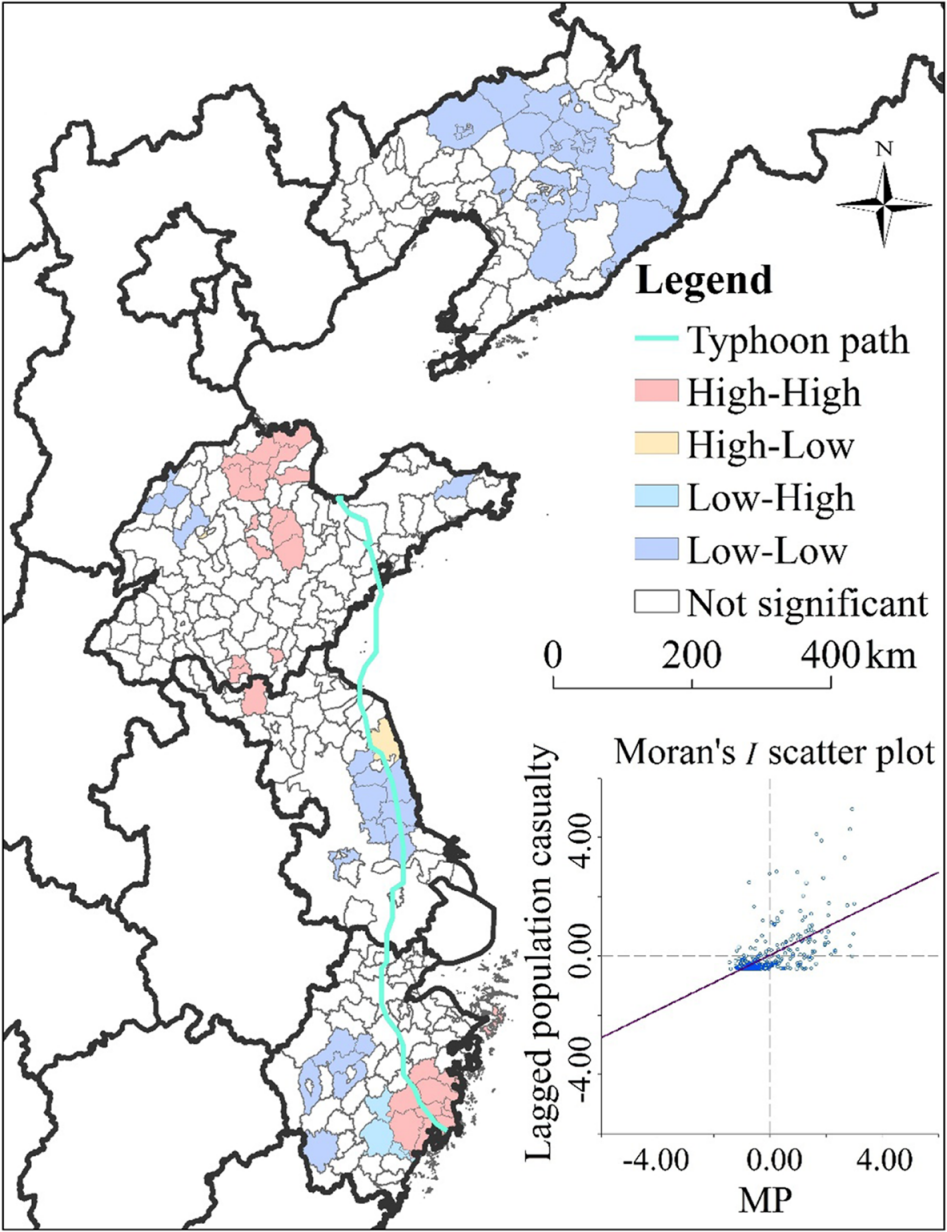
Map showing the distribution of clusters of precipitation and population casualty, with the Moran’s *I* scatter plot (The administrative map in the figure was obtained from the Resource and Environment Data Cloud Platform (http://www.resdc.cn))

Bivariate local spatial association analysis was conducted based on the dominant factor (maximum precipitation) and the population casualty rate attributable to super typhoon Lekima. The results showed that the local Moran’s *I* value of the population casualty rate was 0.46 (*p* < 0.001), indicating that maximum precipitation and population casualty rate had a significant and positive spatial correlation. Based on the results of Moran’s *I* scatter plot, counties in the four quadrants are plotted in Fig. 5. For counties located in the first quadrant, a positive correlation was found between high population casualty rate and severe precipitation. For counties located in the third quadrant, a positive correlation was found between low growth of population casualty rate and low precipitation. Figure 5 shows the spatial clustering of the two variables and counties where the spatial correlation of the two variables is significant. High-high spatial clusters (hot spots) of precipitation and population casualties were observed in counties located in southeastern Zhejiang and northern Shandong. Low-low spatial clusters (cold spots) of precipitation and population casualties were observed in counties located in northern Liaoning and eastern Jiangsu.

## Discussion

Comprehensively considering various hazard factors, sensibility of disaster environments, vulnerability of hazard-affected bodies, and the combined effects of disaster prevention and reduction capacity [13, 21, 25, 34], the determinant powers of natural and socioeconomic factors and their interactive effects on the population casualty rate attributable to super typhoon Lekima were quantified. Then, the spatial pattern of the population casualty rate under the influence of the dominant factor (maximum precipitation) was determined using the LISA model to identify hot and cold spots. The results showed that both natural and socioeconomic factors significantly affect the population casualty rate. Additionally, among all the interactive effects of the selected influencing factors, the interactive effects between maximum precipitation and ratio of brick-wood houses, maximum precipitation and ratio of steel-concrete houses, and maximum precipitation and number of health technicians were the strongest. High-risk areas of high population casualty rate attributable to super typhoon Lekima were mainly distributed in the southeastern counties of Zhejiang and northern Shandong, which suffered the most severe precipitation induced by super typhoon Lekima, while low-risk areas are mainly distributed in northern Liaoning and eastern Jiangsu.

It is generally and globally acknowledged that severe precipitation and strong wind are the main mechanisms of typhoons releasing energy, and these factors largely lead to population casualties and property losses [35–37]. Similarly, in this study, the results showed that maximum precipitation was the dominant factor affecting the population casualty rate attributable to super typhoon Lekima, with a *q* value of 0.56. This implies that severe precipitation has a significant impact on the population casualty rate, which is consistent with the findings of previous research. For example, Hu et al. demonstrated that extreme precipitation can cause flood disasters and contributes towards increasing the exposure of populations and assets [13]. Similarly, Lin et al. showed that heavy precipitation and floods caused by tropical cyclones have caused huge population and economic losses worldwide [38]. These studies indicated that severe precipitation is a key phenomenon of typhoon energy release and one of the main factors causing population casualties and property losses. Moreover, the interactive effects of maximum precipitation and other factors, such as ratio of brick-wood houses, ratio of steel-concrete houses, and number of health technicians, had a strong impact on the population casualty rate, indicating that heavy precipitation will not only have a great impact on the population casualty rate, but also significantly increase the impact with other factors on the house collapse rate. This may be because heavy precipitation could cause secondary disasters, such as floods and mudslides, which lead to major disasters in coastal areas [39], and threaten the safety of human life and property. Moreover, areas with developed economies and dense populations are highly sensitive to disasters, such as floods, rainstorms, and raised water levels. Therefore, the interactive effects of precipitation and socioeconomic factors could increase the magnitude and intensity of typhoon disasters in such regions [40–42].

Strong wind was also an important and non-negligible factor in relation to the population casualty rate attributable to super typhoon Lekima. In this study, the results showed that maximum wind speed has a high determinant power, with a *q* value of 0.45, showing that severe wind has a significant impact on the population casualty rate, which is consistent with the findings of previous research. For example, Nigusse et al. found that strong wind speed induced by typhoons would increase the damage to life and property [18]. Similarly, Li et al. reported that the strong wind speed induced by a typhoon had a significant impact on human life and property [43]. Typhoon disasters may be mainly driven by heavy precipitation and strong wind; the associated water flow and wind would erode or destroy structures or components of houses and further aggravate the collapse of houses, leading to population casualties and serious economic losses.

In addition to natural factors, socioeconomic factors also had an important impact on population casualty rate attributable to super typhoon Lekima. For example, number of health technicians, number of health beds, per capita GDP and population density (*q* values of 0.35, 0.27, 0.16 and 0.14, respectively), which are generally considered to be important factors affecting disaster assessment, would increase the impact intensity of typhoon disasters, especially in the coastal areas, consistent with the results of other previous studies. Ying et al. reported that factors of the ability of disaster prevention and reduction, such as per capita GDP, can reflect the resilience of a region against typhoons [44]. Similarly, Hu et al. showed that population density has a significant impact on population casualties affected by severe precipitation and secondary disasters caused by typhoon disasters [13]. In addition, Wang et al. pointed out that densely population and high economic level will further increase the extent of damages caused by typhoon disasters [45]. These studies showed that rapid urbanization has promoted higher economic growth and attracted a large number of people, resulting in the accumulation of population. In other words, the disaster losses caused by typhoons will further increase as the economic level increases, because cities with high economic levels are accompanied by an increase in population density, with increased concentration of both roads and buildings in metropolitan areas [18]. Therefore, the impact of natural disasters in such areas will increase or expand to some extent.

The interactive effects between natural and socioeconomic factors were all enhanced, that is, the determinant power of two factors were greater than that of single factor, as revealed by the GeoDetector analysis. For instance, the values of *q* (maximum precipitation ∩ ratio of brick-wood houses), *q* (maximum precipitation ∩ ratio of steel-concrete houses) and *q* (maximum precipitation ∩ number of health technicians) were the highest (*q* = 0.72), greater than those on individual factors; this implies that the interactive effects of the factors presented a significant increase over the effects of individual factors on the population casualty rate attributable to super typhoon Lekima. Notably, in recent years, the ecological environment and human living conditions have changed dramatically due to rapid urbanization [46, 47]. The interactive effects between natural and socioeconomic factors will amplify the impact of natural disasters to a certain extent, and the factors will enhance the effects of each other on the population casualty rate.

Specifically, the risk of typhoon disasters is affected by various factors, including meteorology, emergency management, socioeconomic, and physical effects. This study provides a new perspective on study of typhoon disasters by analyzing the interactive impacts of factors, which can provide a certain scientific basis and make contributions for policy formulation. For example, when formulating policies, not only the impact of a single factor, but also the interactive impacts of factors on disaster risk should be considered, which would be helpful to have more concrete insights into what are the typhoon disasters could look like and make contribution for policies and strategies to more fully consider and integrate the interactions of multiple factors to enhance the capacity of disaster response efforts. Moreover, in the process of specific implementation, the risk assessment of typhoon disasters needs to consider these factors comprehensively, so as to compare the typhoon disaster risks of different regions, provide reference for the resource allocation and high-level planning of decision-making departments, and improve the public’s awareness and understanding of typhoon disasters. It is undeniable that cities are the common spatial carrier of typhoon disasters and influencing factors, and none of the factors exist alone. Therefore, further research should pay more attention to the comprehensive interactive impacts of factors on typhoon disasters.

Furthermore, hot and cold spots of population casualty rate in this study were revealed by LISA. Hot spots were mainly located in southeastern Zhejiang and northern Shandong, indicating that these regions experienced significantly severe population casualties. Therefore, these regions should receive more attention during typhoon events. In contrast, cold spots were mainly located in northern Liaoning and eastern Jiangsu, indicating that these regions experienced a significantly low population casualty rate. These results indicate that, in addition to natural factors, the socioeconomic level also has a certain intervention effect on the outcome of disasters, which is consistent with the results of previous research. For example, Zhang et al. reported that heavy precipitation caused by typhoons resulted in a decrease in population mortality with the growth of per capita GDP [48]. Similarly, Hu et al. reported that, with a rise in per capita GDP of $1, flood deaths attributable to typhoons will decrease by 0.41 [13]. The underlying mechanism may be the significant improvements made in disaster prevention infrastructure and the ability to mitigate disasters with socioeconomic development.

This study has some limitations that should be clarified. The first limitation is that only some natural, demographic, and socioeconomic factors were considered as risk factors for population casualty rate, while ignoring environmental factors (such as temperature and specific humidity). Moreover, the natural disaster system is a complex subsystem of the huge earth system, and it is affected by various complicated natural processes and human social activities. The second limitation is that the spatial scale used in this study was at the county level, which may introduce some uncertainties in the study.

## Conclusions

In this study, GeoDetector was used to quantify the determinant powers of natural and socioeconomic factors in Zhejiang, Jiangsu, Shandong, and Liaoning, in order to further examine the dominant factor, and their interactive effects on the population casualty rate attributable to super typhoon Lekima. Then, the LISA method was used to reveal the spatial pattern of population casualty rate under the influence of the dominant factor. The results showed that hot spots were mainly distributed in southeastern Zhejiang and northern Shandong, while cold spots were mainly distributed in northern Liaoning and eastern Jiangsu. Both natural and socioeconomic factors significantly affected the population casualty attributable to super typhoon Lekima. Notably, with rapid urbanization, the impact of socioeconomic factors has been receiving increasing attention in recent years, which further illustrates that the spatial heterogeneity of disaster losses is closely related to socioeconomic factors. Moreover, the interactive effects between natural and socioeconomic factors had stronger impact on the population casualty attributable to the super typhoon Lekima. These findings provide deeper insight into the impact mechanism of typhoon disasters. With better understanding of the mechanism, information can be collected and evaluated more scientifically and reasonably, and the impact factors of the spatial heterogeneity of disaster losses can be understood in greater detail. These results imply that more specific strategies are required for different regions to prevent, control, and allocate resources in order to enhance their disaster response capabilities and reduce potential losses caused by natural disasters.

## Data Availability

The datasets used and/or analyzed during the current study are available from the corresponding author on reasonable request.

## Abbreviations

LISA: The local indicator of spatial association model
RS: Ratio of steel-concrete houses
RC: Ratio of brick-concrete houses
RB: Ratio of brick-wood houses
PD: Population density
PG: Per capita gross domestic product
PT: Proportion of tertiary industry
NB: Number of health beds
NT: Number of health technicians
MP: Maximum precipitation
MW: Maximum wind speed
SP: Slope
EV: Elevation
